# A ^1^H NMR Investigation of the Interaction between Phenolic Acids Found in Mango (*Manguifera indica cv Ataulfo*) and Papaya (*Carica papaya cv Maradol*) and 1,1-diphenyl-2-picrylhydrazyl (DPPH) Free Radicals

**DOI:** 10.1371/journal.pone.0140242

**Published:** 2015-11-11

**Authors:** Luis M. López-Martínez, Hisila Santacruz-Ortega, Rosa-Elena Navarro, Rogerio R. Sotelo-Mundo, Gustavo A. González-Aguilar

**Affiliations:** 1 Departamento de Investigación en Polímeros y Materiales (DIPM), Universidad de Sonora, Calle Rosales y Blvd. Luis Encinas s/n, Col. Centro, Hermosillo, Sonora, 83000, México; 2 Centro de Investigación en Alimentación y Desarrollo (CIAD), A.C. Carretera a Ejido La Victoria Km 0.6, Hermosillo Sonora, 83000, México; UMR INSERM U866, FRANCE

## Abstract

The benefits of phenolic acids on human health are very often ascribed to their potential to counteract free radicals to provide antioxidant protection. This potential has been attributed to their acidic chemical structure, which possesses hydroxyl groups in different positions. Phenolic acids can interact between themselves and exhibit an additive, antagonistic or synergistic effect. In this paper, we used ^1^H NMR to analyze the interactions and mechanisms that are present in major phenolic acids found in mango (gallic, protocatechuic, chlorogenic and vanillic acids) and papaya (caffeic, ferulic and *p*-coumaric acids), and the DPPH radical was used to evaluate the effect of the antioxidant mixtures. The interactions were found to occur via hydrogen bonds between the -OH and -COOH groups. Moreover, the phenolic acids exhibit two types of mechanisms for the neutralization of the DPPH radical. According to the results, these two mechanisms are Hydrogen Atom Transfer (HAT) and Single Electron Transfer (SET). The ability of the phenolic acid to neutralize the DPPH radical decreases in the following order in mango: gallic > chlorogenic > protocatechuic > vanillic. Moreover, within the acids found in papaya, the order was as follows: caffeic > *p*-coumaric > ferulic.

## Introduction

The antioxidant capacity of phenolic acids can be enhanced or reduced as they interact within each other or with other compounds. These synergistic or antagonistic effects are not well understood, and the effects occur in plant extracts and drugs because a pure chemical compound is rarely used [1]. Tropical fruits are rich in antioxidants, and among them, phenolic acids are important. We have shown that major phenols that are present in mango had synergistic or antagonistic interactions, which lead to changes in the antioxidant capacity [2]. However, the possible modes of action or mechanism involved in these reactions are not well documented and require further study. Moreover, the antioxidant activity of phenolic acids is generally governed by their chemical structures; the activity improves as the number of hydroxyl (OH) and methoxy groups increase, and the number of OH groups is more important. Thus, caffeic acid is more active than ferulic acid, and ferulic acid is more active than *p*-coumaric acids [3]. Therefore, most authors concluded that the antioxidant capacity of any phenolic compound is determined by the structural elements [4,5], even a large number of publications on this subject have been published [6,7], contradictory results can be achieved depending the technique used to determine the antioxidant potential of these type of molecules.

Generally, the presence of hydroxyl substituents increases the antioxidant capacity. The number and the position of the hydroxyl groups in the structure of the phenols determine the capacity to donate an electron or a hydrogen atom. The main purpose of this study was to investigate the ability of each individual antioxidant to prevent oxidation in food systems, which has not been evaluated even though some studies have evaluated the interactions between different antioxidant compounds [8,9,10,11,12].

Nuclear magnetic resonance (NMR) is a sophisticated and powerful analytical method that has found a variety of applications, such as the identification of structures and molecular interactions. Many studies of the application of NMR to identify phenolic acids in different extracts have been reported [13,14]. The ^13^C NMR technique has been used to understand the antioxidant molecular mechanism of catechins and phenols [15,16,17]. Kawabata *et al*. studied the oxidative products of protocatechuic and gallic esters with DPPH using ^1^H and ^13^C NMR [18]. Other NMR applications involve the study of the scavenging mechanism of catechols against the DPPH radical [19]. Tazaki *et al*. reported a ^1^H and ^13^C NMR study of the formation of *o*-quinone from caffeic acid using NaIO_4_ [20].

The objective of the present work was to use NMR to evaluate the antioxidant properties of the individual and combined compounds at specific molar concentrations of the major phenols that are present in mango and papaya fruit.

## Materials and Methods

Commercial standards of caffeic, ferulic, *p*-coumaric, chlorogenic, gallic, protocatechuic and vanillic acids (Sigma-Aldrich, Toluca, México) and a stable free radical, 1,1-diphenyl-2-picrylhydrazyl (DPPH), were used for all experiments.

### Method

The ^1^H NMR spectra of the individual and mixed standard phenolic acids with or without the stable free radical, DPPH, were obtained using a Bruker Avance 400 spectrometer that operates at 400 MHz. The chemical shifts are expressed as δ ppm values using TMS as the internal standard. All spectra were taken in DMSO-d_6_.

### Sample preparation for the NMR measurement

Solutions were prepared at a concentration of 5 mM in DMSO-d_6_ for each phenolic acid or mixture of phenolic acids using a ratio of 1:1 and 0.5 mL of this solution was placed into a NMR quartz tube to collect the spectra. The mixtures of the phenolic acids that were studied included the following: gallic and protocatechuic acids; gallic and chlorogenic acids; gallic and vanillic acids; protocatechuic and chlorogenic acids; protocatechuic and vanillic acids; chlorogenic and vanillic acids; gallic, protocatechuic and chlorogenic acids; gallic, protocatechuic and vanillic acids; gallic, chlorogenic and vanillic acids; protocatechuic, chlorogenic and vanillic acids; gallic, protocatechuic, chlorogenic and vanillic acids; caffeic and *p*-coumaric acids; caffeic and ferulic acids; *p*-coumaric and ferulic acids; and caffeic, *p*-coumaric and ferulic acids.

After the ^1^H NMR spectrum of each sample was obtained, an antioxidant capacity measurement was performed by adding DPPH to the NMR tube [21]. The addition of DPPH was performed using a 1:1 ratio with respect to the concentration of the solution of the phenolic acids, either individually or as mixtures. All of the mixtures were allowed to stand for 3 min at room temperature, and after the purple color of the DPPH faded, they were subjected to ^1^H NMR analysis. The spectra were further analyzed using MESTREC software (MesrReNova v9.0.1–13254, Mestrelab Research S.L., Spain).

## Results and Discussion

**Fig 1:**
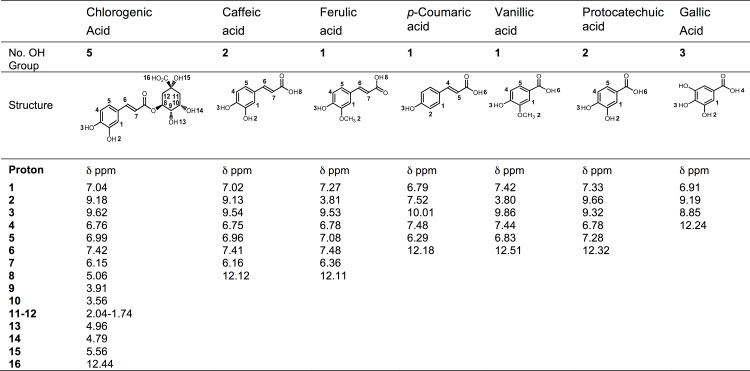
Structures of the phenolic acids found in mango and papaya fruit. The number of OH groups and the chemical shifts, δ (ppm), obtained using proton NMR in DMSO-d^6^ for each acid.

The ^1^H NMR spectra of all of the compounds were obtained in DMSO-d_6_ at a 5 mM concentration. Fig 1 presents the chemical shift of each proton for each phenolic acid studied. The phenolic acids included in this study are the major compounds of papaya (caffeic, ferulic and *p*-coumaric) and mango (gallic, chlorogenic, protocatechuic and vanillic acids) [2, 22].

**Fig 2:**
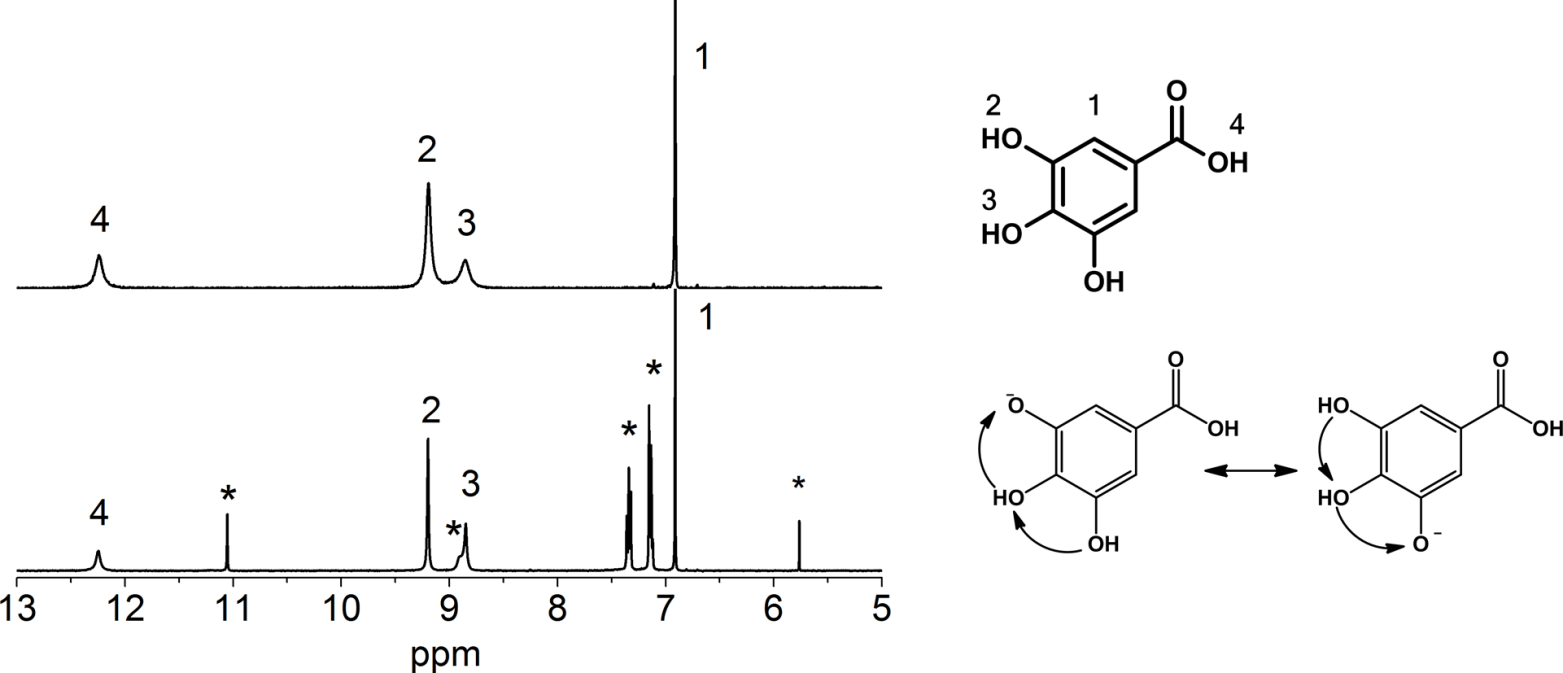
^1^H NMR spectra of gallic acid (top) and gallic acid + DPPH radical (bottom) in DMSO-d_6_. The signals labeled by an *asterisk* are attributed to the DPPH radical.

Fig 2 Shows the ^1^H NMR spectra of gallic acid in DMSO-d_6_ before and after reaction with DPPH. The gallic acid spectrum shows the characteristic hydroxyl protons at 8.85 and 9.19 ppm and the acid group at 12.15 ppm. The hydroxyl and carboxylic protons have broad signals in the upper spectra, which are indicative of the interaction with other -OH groups that are possibly water remnants [23] that are found between 9 and 11 ppm.

Upon reaction, the gallic acid signals sharpen, and new peaks arise from DPPH, which are identified with an asterisk. This sharpening may be due to the loss of one of the aromatic hydroxyls upon reaction with DPPH, and the remaining protons are delocalized because they are in the *ortho* configuration. After the reaction, a decrease in the integral of the -OH groups was observed upon the comparison with the integral of the sign of the aromatic protons that are labeled with 1 (S1 Fig). We observed that DPPH was neutralized, and its color changed faster from purple to brown. However, no new signals were detected with NMR, indicating that no quinone was formed. Charisiadis *et al*. reported a significant reduction in the bandwidth of the -OH protons of caffeic acid and hydroxytyrosol by adding picric acid, which resulted in a minimum -OH proton exchange rate [24].

**Fig 3:**
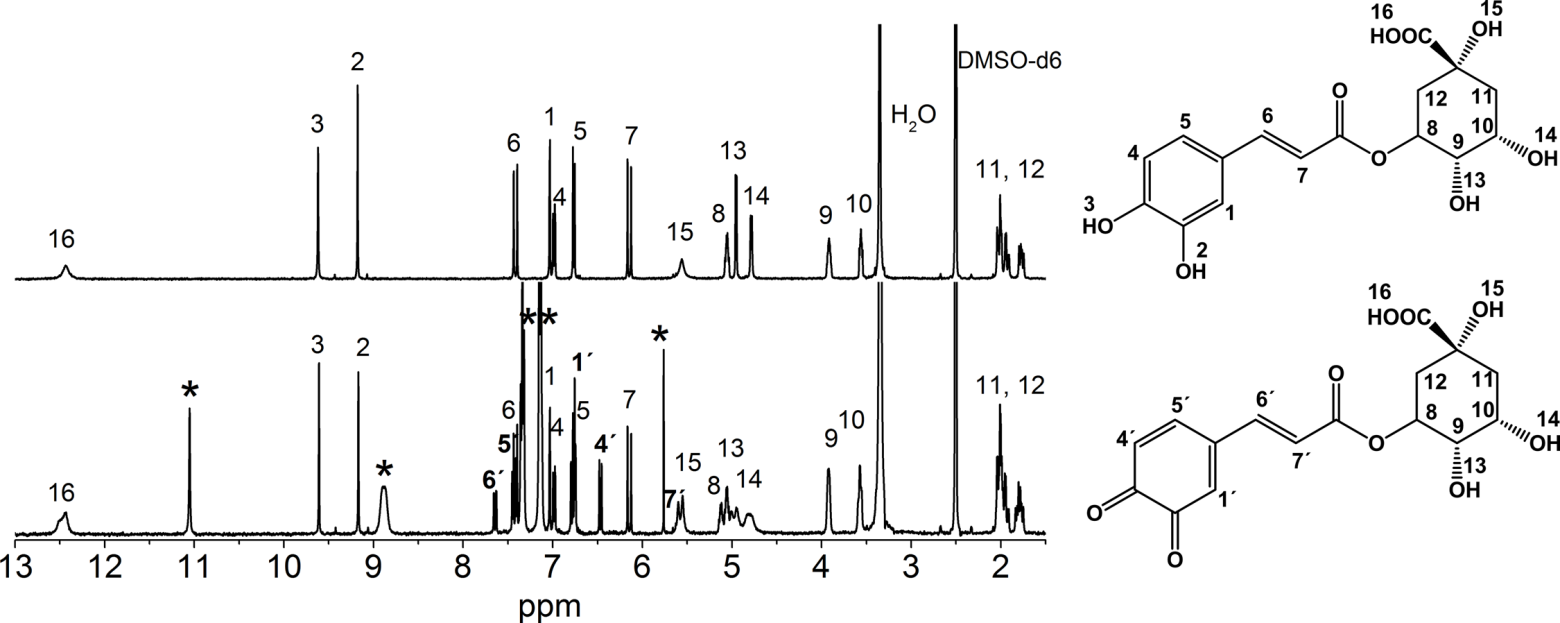
^1^H NMR spectra of chlorogenic acid (top) and chlorogenic acid + °DPPH radical (bottom) in DMSO-d^6^. The signals with an apostrophe are due to the quinone formed upon the neutralization of the °DPPH radical. The signals labeled with an *asterisk* are attributed to the °DPPH radical.

The spectrum of chlorogenic acid was more complex because of its chemical structure (Fig 3). This phenolic acid has the acid group in the non-aromatic ring, which changes its chemical properties. Only two broad peaks were observed (protons 15 and 16) that correspond to the carboxylic and hydroxyl groups at position of C5 in the non-aromatic group.

This signals appear only using DMSO-d_6_ because those protons are quickly exchanged in D_2_O and are not observed. Upon reaction with DPPH, peak 15 sharpens, which is due to the hydroxyl group. For this compound, besides the change in color due to the DPPH neutralization, we observed the appearance of new signals in the NMR spectra that are consistent with the formation of a quinone. Therefore, chlorogenic acid is now a quinone derivative, leading to changes in the unsaturated cinnamic arm and the displacement of protons in the non-aromatic ring. DPPH was identified, and its protons are labeled with an asterisk. The signals of the chlorogenic acid-quinone protons are at 6.74(H1), 6.46(H4), 7.65(H5) ppm, whereas the H6 signal appears at 7.43 ppm and the H7 signal is at 6.78 ppm. Additionally, changes in the signals of all of the protons of the non-aromatic ring were observed as a consequence of the quinone formation. These effects produced a downfield shift of the signals due to a change in their environment. The effect was most evident in the signals of protons 13 and 14, which correspond to the -OH groups at 4.95 and 4.78 ppm, respectively. Before the reaction was performed, the signals appeared as doublets due to their links with protons that are labeled 9 and 10, respectively, with values of J = 6 Hz. Similar changes were observed for the caffeic and protocatechuic acids. The same analysis was performed for all of the other phenolic acids and their mixtures.

DPPH reacted with the caffeic, *p*-coumaric, protocatechuic, gallic and chlorogenic acids, as observed by the rapid color change of the solution from purple to brown. We found that ferulic and vanillic acids did not present a color change after contact with the DPPH radical for several hours. This behavior was observed in previous studies that evaluated the antioxidant capacity of these acids using UV-Vis [2]. Nonetheless, this does not imply that the chemical structure of the phenolic acid is changing; it only suggests that DPPH oxidizes via neutralization.

The reaction of phenolic acids with radicals can lead either to a quinone derivative or their original chemical structure. In this work, the chlorogenic, caffeic and protocatechuic acids formed quinones via the reaction with the stable DPPH radical, and the gallic and *p*-coumaric acids did not change their structures although all of them neutralized DPPH.

There are two mechanisms for the antioxidant neutralization of the DPPH radical using the phenolic acids: Hydrogen Atom Transfer (HAT) and Single Electron Transfer (SET). Table 1 presents the antioxidants studied in this work and the mechanism that occurs for each phenolic acid.

**Table 1:** Proposed mechanisms for the neutralization of the DPPH radical with the antioxidant compounds of mango and papaya.

Phenolic Acid	Mechanism	Reaction with DPPH
Gallic	HAT	(+)
Protocatechuic	SET	(+)
Chlorogenic	HAT, SET	(+)
Vanillic	(-)	(-)
Caffeic	SET	(+)
p-Coumaric	HAT	(+)
Ferulic	(-)	(-)

HAT refers to the loss of a proton to the radical and to the stabilization of the charge by nearby groups. This mechanism produces no change in the chemical structure because the rapid proton exchange is observed as a narrowing of the proton signals [16,20]. This is not an indication that the reaction not occurred, merely that the changes that take place during the stabilization of the system are too fast and impossible of obtain an accurate measurement by the NMR technique. In contrast, the SET mechanism leads to a loss of a proton and a rearrangement of the structure, usually with the formation of a double bond and quinone derivatives. In this mechanism, the structure of the phenolic acid is clearly changed and, as a consequence, the proton signals that are detected by ^1^H NMR also change [16,20]. The possible mechanisms of action of gallic and chlorogenic acid as antioxidants is shown in S2 Fig.

No new signal appears after the reaction of gallic acid with the DPPH radical in the ^1^H NMR spectra. This indicates that gallic acid is stabilized as a radical, and it is not converted to a *o*-quinone moiety. Therefore, a HAT mechanism occurs, similar to that observed with the *p*-coumaric acid. A similar behavior is present in the tea polyphenols[16] In the ^1^H NMR spectra of the protocatechuic, chlorogenic and caffeic acids after the reaction with DPPH, the signals of the protons, H1 and H4, shift upfield, and the H5 shifts downfield, indicating the presence of the *o*-quinone of the ring. This behavior indicates a SET mechanism [16,20].

To determine whether there is an interaction between the different phenolic acids, mixtures of them were prepared, as shown in Tables 2 and 3. Although DMSO-d_6_ solvent can competitively interact with the phenolic acids, this solvent allows to observe the different interactions between the protons of the OH and COOH groups, this interaction was no observed when the protic solvent-ethanol-d_6_ was used. Tables 2 and 3 displays the different combinations of the phenolic acids present in mango and papaya, respectively.

**Table 2:** Binary and ternary combinations of phenolic acids that are present in mango and their ability to neutralize the °DPPH after 3 minutes of reaction.

		Antioxidant	Phenolic acids involved in neutralization			
Mixture phenolic acid + DPPH	Mechanism	capacity relative to weighted average	Gallic	Protocatechuic	Chlorogenic	Vanillic
Gallic and Protocatechuic	HAT-SET	SYN +++	✓			
Gallic and Chlorogenic	HAT-SET	SYN ++	✓		✓	
Gallic and Vanillic	HAT	ANT +	✓			
Protocatechuic and Chlorogenic	SET-SET	SYN +++			✓	
Protocatechuic and Vanillic	SET	SYN +		✓		
Chlorogenic and Vanillic	SET	SYN +			✓	
Gallic, Protocatechuic and Chlorogenic	HAT-SET-SET	SYN +	✓		✓	
Gallic, Protocatechuic and Vanillic	HAT-SET	SYN ++	✓			
Gallic, Chlorogenic and Vanillic	HAT-SET	SYN +++	✓		✓	
Protocatechuic, Chlorogenic and Vanillic	SET-SET	ANT +			✓	
Gallic, Protocatechuic, Chlorogenic and Vainillic	HAT-SET-SET	SYN ++	✓		✓	

SYN-synergism, the actual antioxidant capacity of the mixture is greater than the weighted average of the antioxidant capacity of each phenolic acid. ANT–antagonism, the actual antioxidant capacity of the mixture is less than the weighted average of the antioxidant capacity of each phenolic acid. +, ++, +++ -indicates the intensity of increase of the antioxidant activity according the phenol combination used.

**Table 3:** Binary and Ternary combinations of phenolic acids that are present in papaya and their ability to neutralize the DPPH after 3 minutes of reaction.

		Antioxidant	Phenolic acids involved in neutralization		
Mixture phenolic acid + DPPH	Mechanism	capacity DPPH %	Caffeic	*p*-Coumaric	Ferulic
Caffeic and p-Coumaric	SET-HAT	78	✓		
Caffeic and Ferulic	SET	92	✓		
*p*-Coumaric and Ferulic	HAT	58		✓	
Caffeic, *p*-Coumaric and Ferulic	SET-HAT	82	✓		

The different antioxidant mixtures were prepared to a final concentration of 5 mM for each compound in the solution. Once we prepared the different solutions, the ^1^H NMR spectra were measured, analyzed and compared with their individual spectra.

Generally, the different combinations of phenolic acids that we studied exhibited -OH group signals that were shifted downfield and widened. A similar behavior was observed with the -COOH group. For this reason, we can assume that hydrogen bond interactions between the different molecules in the mixture of phenolic acids occurred.

To determine which of the acids that was present in each mixture reacts first during the DPPH neutralization, an aliquot of 50 μL of DPPH radical solution at a concentration of 50 mM was added to each tube. The tubes were stirred, and the ^1^H NMR spectra was obtained after 3 minutes. All of the solutions changed color from purple to brown, indicating that the neutralization of DPPH occurred.

Table 2 displays the results obtained for the mixtures of phenolic acids that are present in mango, and it also shows which compound is responsible for the DPPH color change, according to the observed changes in the ^1^H NMR spectra. For all of the spectra of the solutions containing chlorogenic acid, we observed the formation of the quinone group in the chlorogenic acid upon the neutralization of the DPPH radical.

**Fig 4:**
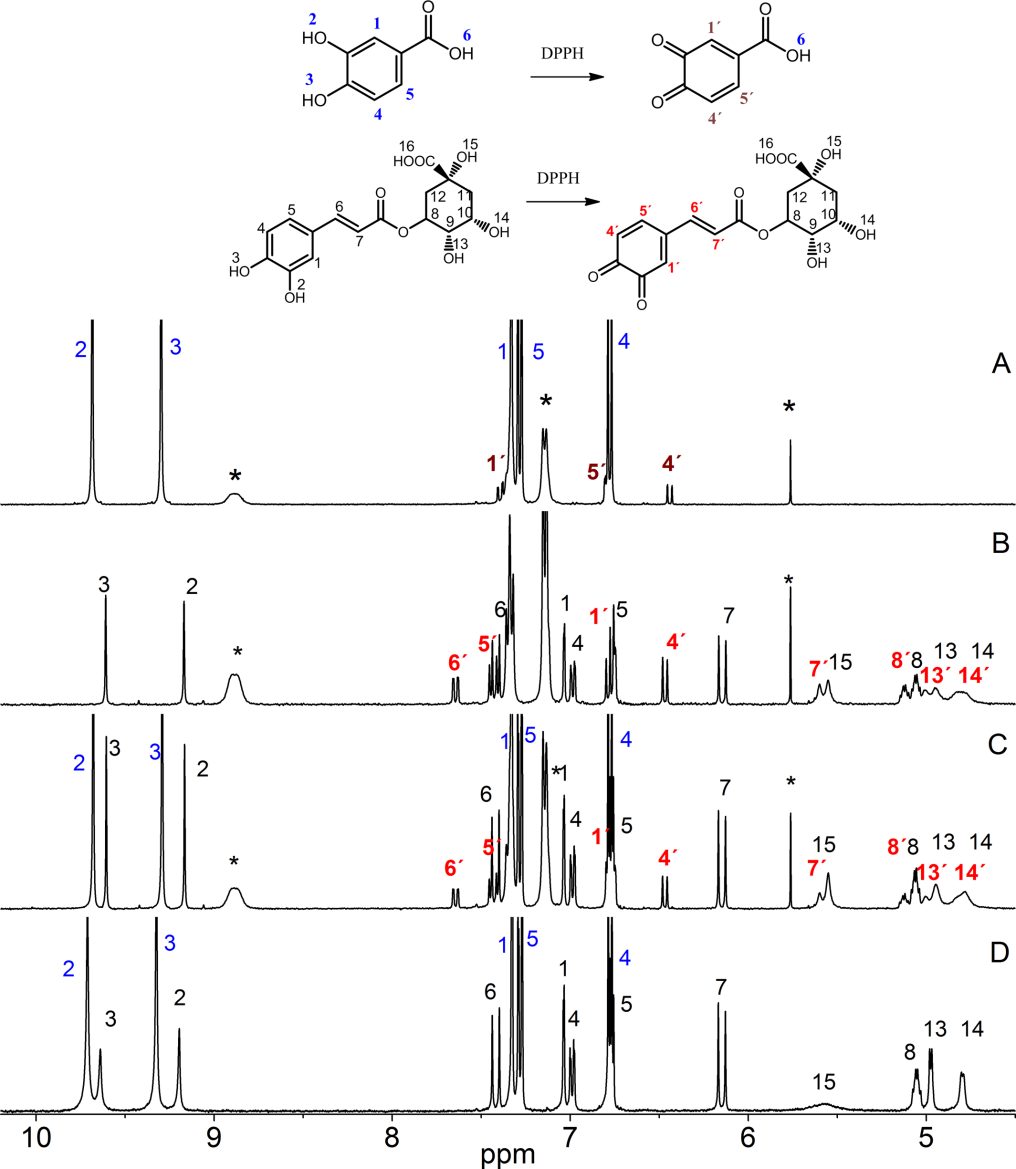
^1^H NMR spectra representing the competitive ability of protocatechuic acid vs. chlorogenic acid to neutralize the °DPPH radical in DMSO-d^6^: (A) protocatechuic acid + the °DPPH radical, (B) chlorogenic acid + the °DPPH radical, (C) mixture of the two phenolic acids + the °DPPH radical and (D) mixture of the two phenolic acids. The signals labeled with an *asterisk* are attributed to the °DPPH radical.

Fig 4 displays the spectra representing the competitive ability of the mixture of chlorogenic and protocatechuic acids to neutralize the DPPH radical. Both molecules exhibited the same mechanism of action, which occurred via electron transfer with the formation of the quinone group. As shown in the figure, the presence of new signals after the reaction with the DPPH radical corresponds to quinone group formation in the chlorogenic acid. In all of the other mixtures in which the chlorogenic acids were present, the formation of quinone was observed.

In the competitive study of the binary mixtures of gallic acid-vanillic acid and gallic acid-protocatechuic acid, we found out that gallic acid was the most reactive. In the mixture of the gallic and vanillic acid, the gallic acid was responsible for the color change because there was no response by the DPPH radical due to the vanillic acid. However, in the spectra of the mixture of gallic and protocatechuic acid, we did not observe any signal for the formation of the quinone group.

Moreover, for the mixture of the protocatechuic and vanillic acid, the compound responsible for the color change was protocatechuic acid, which presented a new signal in the spectrum that corresponds to the formation of quinone due to the protocatechuic acid.

**Fig 5:**
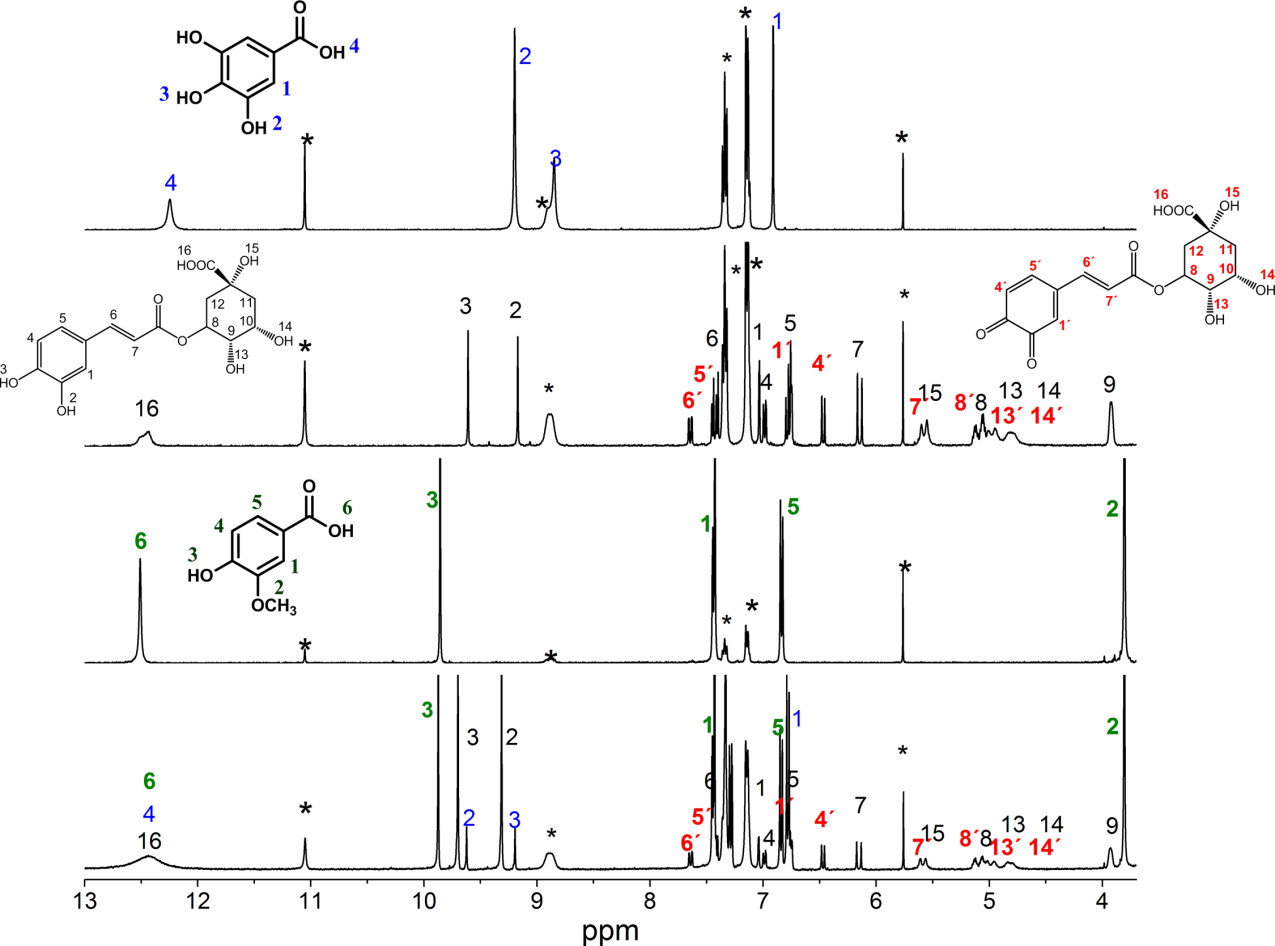
^1^H NMR spectra representing the competitive ability of gallic acid, chlorogenic acid and vanillic acid to neutralize the °DPPH radical in DMSO-d^6^: (A) gallic acid + the °DPPH radical, (B) chlorogenic acid + the °DPPH radical, (C) vanillic acid + the DPPH radical and (D) mixture of the three phenolic acids + the °DPPH radical. The signals labeled with an *asterisk* are attributed to the °DPPH radical.

Fig 5 shows the spectra of the individual and the ternary mixture of chlorogenic, gallic and vanillic acids after reacting them with the DPPH radical. However, when we compared the individual spectra of each phenol with the spectrum of the mixture, we clearly observed the presence of new signals that correspond to the formation of the quinone group due to the chlorogenic acid. Thus, we assume that chlorogenic acid is the first compound to react in this mixture. The same behavior was observed for the mixtures of gallic, protocatechuic and chlorogenic acids and protocatechuic, chlorogenic and vanillic acids. For the mixture of gallic, protocatechuic and vanillic acids, the first compound in the neutralization of DPPH was gallic acid. The capacity of the phenolic acids that are present in mango to neutralize the DPPH decreases in the following order: chlorogenic > gallic > protocatechuic > vanillic. In our study by UV-Vis, the antioxidant response was gallic> chloragenic>protocatechuic>vanillic, possibly the reversal of the response observed between gallic and chlorogenic acids can be attributed to not be detected by ^1^H NMR technique species formed by HAT mechanism. Gallic acid probably reacts faster than chlorogenic acid but the high sensibility of the technique used in this work does not allow it to detect.

Table 3 displays the results obtained for the mixtures of phenolic acids that are present in papaya. For all of the solutions of different combinations, caffeic acid was responsible for the neutralization of DPPH radical because the presence of new signals corresponding to the formation of the quinone group in the molecule of caffeic acid was observed in the ^1^H NMR spectra. For the mixture of *p*-coumaric and ferulic acids, *p*-coumaric was responsible for the color change. The capacity of the phenolic acids that are present in papaya to neutralize the DPPH decreases in the following order: caffeic > *p*-coumaric> ferulic.

## Conclusions

The phenolic acids present in mango and papaya exhibit a good antioxidant activity. The ^1^H NMR analysis using DMSO-d_6_ as the solvent has reinforced the evidence that was reported previously by other authors using less sophisticated techniques. The DMSO-d_6_ allows us to observe the intramolecular and intermolecular interactions that occur between individual phenols and their effects on the antioxidant capacity of these compounds. We observed the interaction between the phenolic acids alone and when they are combined. Such interactions appear to occur via hydrogen bonding between the -OH and -COOH groups. The ^1^H NMR spectra show that the phenolic acids that are found in mango and papaya, such as gallic and p-coumaric acids, exhibit the HAT mechanism for neutralization of the DPPH radical. The protocatechuic, chlorogenic and caffeic acids exhibited the SET mechanism for neutralization of the DPPH radical. From the acids that are found in mango, such as the gallic, protocatechuic, chlorogenic and vanillic acids, chlorogenic acid showed the most efficient response to the elimination of the DPPH radical followed by the gallic and protocatechuic acids. However, vanillic acid exhibited poor activity. Moreover, within the acids found in papaya, the abilities to neutralize the DPPH decrease in the following order: caffeic > *p*-coumaric > ferulic.

## Supporting Information

S1 Fig Integration signals ^1^H NMR spectra of gallic acid (top) and gallic acid + °DPPH radical (bottom) in DMSO-d_6_ are shown in this supplementary figure. (DOCX)

S2 Fig Neutralization mechanisms of a) gallic acid and b) chlorogenic acid to stabilize the °DPPH radical. (DOCX)
