# Supplementary material for: A 1H NMR Investigation of the Interaction between Phenolic Acids Found in Mango (Manguifera indica cv Ataulfo) and Papaya (Carica papaya cv Maradol) and 1,1-diphenyl-2-picrylhydrazyl (DPPH) Free Radicals
Source: PLoS One. 2015 Nov 11;10(11):e0140242. doi: 10.1371/journal.pone.0140242 (PMC4641691; doi:10.1371/journal.pone.0140242)
Supplement: S1 Fig — (DOCX) [file pone.0140242.s001.docx]

Supplementary Material


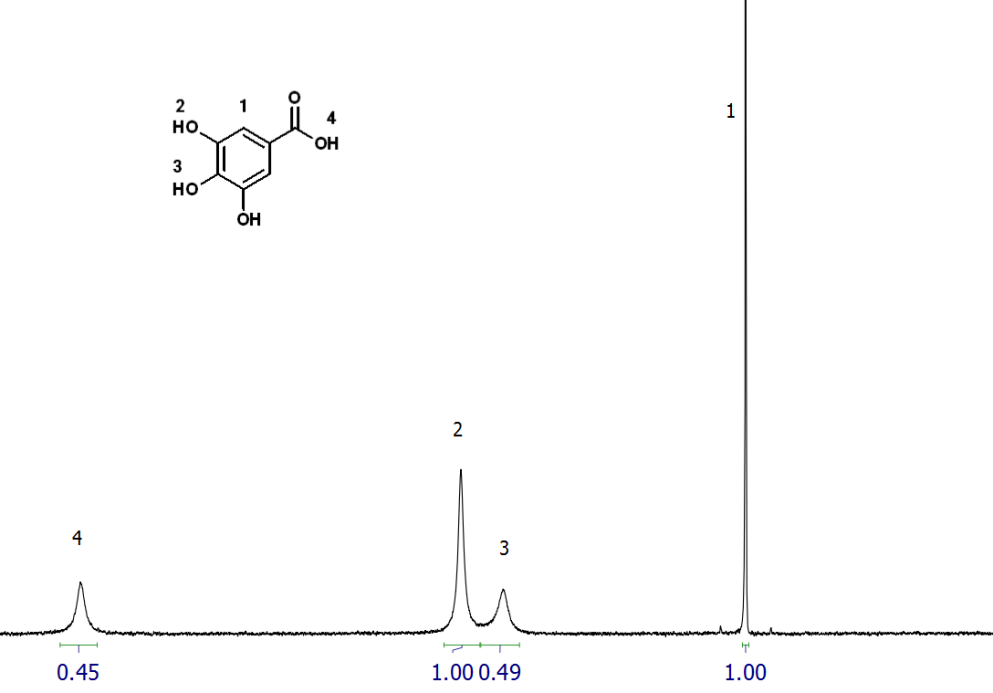


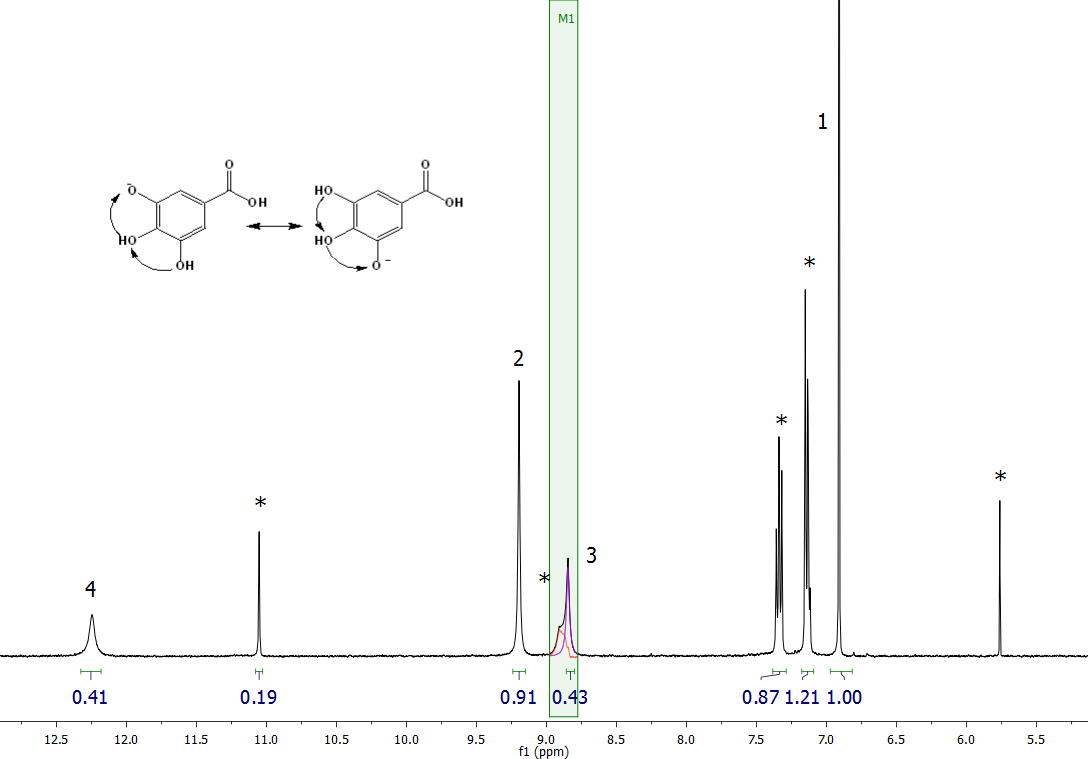


S1 Fig. 1. Integration signals ^1^H NMR spectra of gallic acid (top) and gallic acid + ºDPPH radical (bottom) in DMSO-d_6_ are shown in this supplementary figure.
