# Supplementary material for: A 1H NMR Investigation of the Interaction between Phenolic Acids Found in Mango (Manguifera indica cv Ataulfo) and Papaya (Carica papaya cv Maradol) and 1,1-diphenyl-2-picrylhydrazyl (DPPH) Free Radicals
Source: PLoS One. 2015 Nov 11;10(11):e0140242. doi: 10.1371/journal.pone.0140242 (PMC4641691; doi:10.1371/journal.pone.0140242)

A)

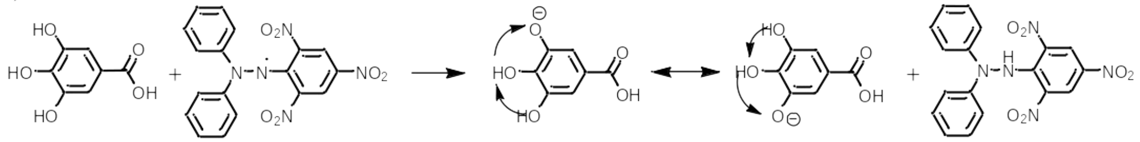

B)

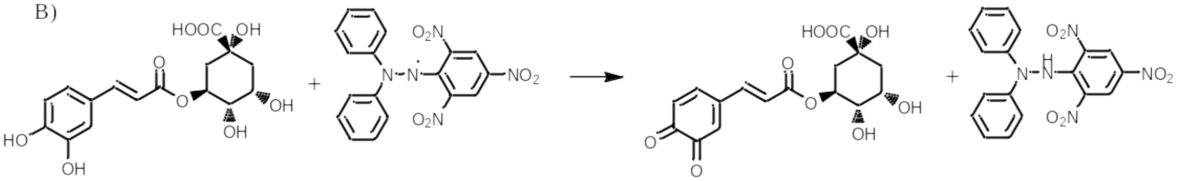

Supplement: Supplementary file 2 [file pone.0140242.s002.docx]
